# Life or Livelihood? Mental Health Concerns for Quarantine Hotel Workers During the COVID-19 Pandemic

**DOI:** 10.3389/fpsyg.2020.02168

**Published:** 2020-09-15

**Authors:** Yi-Man Teng, Kun-Shan Wu, Kuan-Ling Lin

**Affiliations:** ^1^College of Modern Management, Yango University, Fuzhou, China; ^2^Department of Business Administration, Tamkang University, Taipei, Taiwan; ^3^College of Child Development and Education, Yango University, Fuzhou, China

**Keywords:** COVID-19, quarantine hotel workers, mental health, quarantine hotel, mental health assistance programs

## Introduction

A novel coronavirus pneumonia, known as COVID-19 (coronavirus disease 2019), spread rapidly around the globe. The worldwide outbreak of pandemic had apprehended more than half of the population of the world that has caused immense damage to human health and economic and social life (Fareed et al., 2020; Shehzad et al., 2020). It paused the majority of global economic activities, causing major impacts on most industries, particularly the hospitality and leisure industry. As a result, hotels, viewed as a declining industry, have suffered a staggering drop in average occupancy (ATOMIZE, 2020). It is not only hotels in Western countries affected (Djeebet, 2020), the Chinese hotel industry has also been negatively impacted by the COVID-19 outbreak (Zhang et al., 2020). A major dilemma is the financial burden of keeping the hotel open vs. many people losing their jobs if it closes. With such severe impacts, the hospitality industry must learn to function in a new way.

With positive cases of COVID-19 escalating, hospitals face problems with overcrowding and insufficient isolation space (Feng and Cheng, 2020). In addition, there is increasing worry from frontline health care workers about contracting the virus and contaminating loved ones (Rosemberg, 2020). A quarantine hotel is one possible solution to address both these issues. In addition, as the COVID-19 pandemic continues, more countries require both citizens and foreign visitors arriving from abroad to enter a mandatory 14-day quarantine period within centralized observation centers or at home. Because of the shortage of quarantine infrastructure, some hotels have been commandeered by governments to be quarantine hotels.

Providing quarantine space is a smart business move for the hospitality industry. Quarantine packages will not only assist individuals (e.g., health care workers) but will also provide some economic relief for the hospitality industry (Rosemberg, 2020). Many hotels are now considering offering quarantine packages, and some have already been put in place in Austria, China, Malaysia, New Zealand, Taiwan, and the United States.

During the COVID-19 pandemic, the hospitality industry and those who make a living within it will face unprecedented changes. The resulting economic stress, isolation, and uncertainty can quickly take a toll on their physical and mental health. When a hotel transforms into the quarantine hotel, employees must decide whether to continue to earn a living while risking their lives or to walk away. It is a very difficult choice for quarantine hotel employees.

There have been numerous articles written in recent months regarding mental health care from a medical perspective (e.g., health care workers) during the COVID-19 pandemic (e.g., Kang et al., 2020; Walton et al., 2020). However, to date, it is difficult to find relevant literature or empirical studies exploring quarantine hotel employees' mental health and coping strategies. This article is more concerned about the new working situation, conditions, and mental health considerations for quarantine hotel staff during the COVID-19 pandemic. The aims of this research are 2-fold: first, to demonstrate the specific changes to roles and workload for the quarantine hotel employees; and second, to protect the quarantine hotel employees' mental health and provide recommendations for hoteliers in order to support their staff.

## Physical Changes of The Quarantine Hotels

The quarantine hotel must provide the basic amenities for centralized observation during the period of mandatory quarantine, good living conditions, and independent rooms. As a quarantine hotel in China, the workload now includes following an operation guide, complying with antiepidemic and disinfection standards, and implementing quarantine services, as follows:

### Antiepidemic Check-In and Check-Out Guide

(1) Receptionists are required to wear personal protective equipment (PPE), including surgical masks, latex gloves, disposable gowns, caps, and eye protection.
(2) Check-in and check-out counters should be in different areas of the hotel to minimize the risk of infection.
(3) Temperatures of new guests must be taken before they enter the lobby.
(4) A receptionist distributes masks, gloves, and thermometers at a different counter. They also need to provide and explain an information sheet about COVID-19.

### Disinfection and Cleaning Guide

(1) Cleaners must wear PPE.
(2) All potentially contaminated surfaces, including elevators, rooms, passages, lobbies, reception desks, public toilets, hotel entrances and exits, door handles, and any other public facilities, should be disinfected using household bleach at least three times a day and recorded.
(3) Wet disinfecting foot pads should be installed at lobby and elevator entrances.
(4) Emphasis should be on strict cleaning and disinfection of air conditioning or fresh air systems, water supply, and drainage equipment.

### Implementing Quarantine Services

(1) Check and record the quarantine guests' body temperatures daily.
(2) Three meals a day are provided using disposable lunch boxes on a trolley outside guest rooms to avoid contact with quarantine guests.
(3) Guest rooms must be fully cleaned and disinfected prior to quarantine guests' check-in or after check-out.
(4) Guest rooms and public areas should have classified bins for collection of wet and dry garbage. The cleaners disinfect, collect, sort, and record the garbage outside the rooms every day.

## Psychological Experiences of The Quarantine Hotel Staff

Although quarantine hotels provide quarantine space for exposed individuals, providing beneficial assistance to the public during this crisis is also likely to yield some financial relief (Rosemberg, 2020). However, the challenge for quarantine hotel staff is not only the increasing workload created by the quarantine hotel operation but also high psychological stress associated with job insecurity, risk of exposure, and contagion for themselves, their friends, and families.

The quarantine hotel employee hosts those who need quarantine infrastructure, medical staff, first responders, and/or hospital patients not suffering from COVID-19 with new and frequently changing protocols and PPE. Communication with quarantine guests during check-in and check-out is made more complicated by PPE, which covers most of the face, while quarantine hotel cleaners' duties have increased by the necessary disinfection of rooms and hotel areas, also with PPE. As the main route of transmission is from respiratory droplets and direct contact with quarantine guests, quarantine hotel staff face a higher risk of infection. Thus, it is important to keep the health and safety of quarantine hotel employees, particularly the cleaners, at the center of operations. These changes may cause quarantine hotel staff to be distressed and/or feel vulnerable. Consequently, this can cause negative impacts to their mental health, which quarantine hoteliers need to aim to mitigate.

## What Can Quarantine Hoteliers Do for Staff?

Employers have a duty to protect the health and safety of their workers. Although temporary quarantine hotels slightly relieve financial pressures caused by the COVID-19 outbreak and contribute to corporate social responsibility (CSR) worldwide, it is possible that quarantine hotel employees are at a greater risk of adverse mental health outcomes owing to physical fatigue, risk of infection, and shortages of PPE.

Because of the increased risk of adverse mental health of staff, the quarantine hoteliers need to support the employees' mental health needs during the quarantine hotel period of operation. In this setting, it is suggested that quarantine hoteliers should develop short- and long-term mental health assistance programs for employees based on the following recommendations.

### Adequate Training

Proper training is essential during and after the COVID-19 pandemic and is viewed as a protective factor against mental health issues (Brooks et al., 2018). Training helps educate employees about necessary behaviors and their importance in preventing the spread of the virus (Hamouche, 2020), for example, training staff in various COVID-19 protective measures including causes, transmission types, symptoms, cleaning, and disinfection safety procedures. It is therefore recommended that hoteliers implement co-development programs to train their employees in preparation for the impact of COVID-19 in the workplace.

### Open Communication Pathway

During the COVID-19 pandemic, communication is crucial in order to reduce the quarantine hotel employees' uncertainty and stress levels (Hamouche, 2020). In this context, it is recommended that hoteliers develop a communication plan to provide employees with clear information about what will happen during the COVID-19 pandemic, for example, daily updates of pandemic information and the quarantine guests' status, what major actions will be taken to operate the hotel, and the potential impact of these actions on the employees' workloads. Furthermore, they should build a two-way communication platform that encourages staff to express their needs and concerns, as this is one of the most useful resources to promote resilience in quarantine hotel employees. Strategies to improve their mental health will inspire staff to believe they are part of a working family, and together they can overcome the challenges presented by the COVID-19 pandemic now and in the future.

### Building a Resilience Model

In the stressful working atmosphere of COVID-19, arousing a positive working environment is compulsory. Quarantine hoteliers can build a resilience model by encouraging supportive interactions between workers via bulletin boards and online and highlight their sense of honor and pride for continuing to work in a high-risk situation. These are significant environmental protective factors of resilience, as they are strongly related to mental health and considered essential components of successful psychosocial adjustment (Shastri, 2013). The more employees who engage with and benefit from the resilience model, the more the working atmosphere will be improved.

### Promoting Organizational Support

Some studies have suggested that inadequate psychological support from employers is a risk factor for their mental health (Brooks et al., 2018; Hamouche, 2020). Employees who have high perceptions of organizational support are less likely to experience long-term physical and psychological health problems, such as depression and anxiety (e.g., Liu et al., 2015; Lei and Chen, 2020). In order to mitigate the potential negative impact of quarantine, fear of infection, and uncertainty on employees, hoteliers need to create a supportive environment in the workplace, for example, using online surveys to assess the scope of mental health problems and to observe the staffs' psychological status. Once mental health symptoms are identified, rapid access to psychological assistance and contingency for time off work should be provided. In addition, employee assistance programs, such as developing online materials for mental health education and counseling, etc., can provide psychosocial support (Liu et al., 2020). The quarantine hotelier can also implement additional benefits and practical support strategies, such as reducing workloads or hours, as well as increasing remuneration.

## Discussion

During the COVID-19 pandemic, the hotel industry faces unprecedented changes. This article is concerned with the risks to quarantine hotel employees' mental health and aims to suggest that quarantine hoteliers develop coping strategies to minimize the negative impact of COVID-19 on their employees' mental health.

To echo the prior study of Qiu et al. (2020), we suggest quarantine hoteliers provide counseling services for psychological first aid through telemedicine for their staff. It also encourages offering the communication pathways for hotel employees of clear communication with a regular update about COVID-19. Further, parallel to the views of Hamouche (2020), to develop the urgent timely mental health care team to offer regular screening psychological anxiety and depression should be performed for patients, as well as quarantine hotel workers. Having the effective preventive mental health measures in the workplace will help to reduce staffs' level of stress. The quarantine hoteliers have the responsibility to protect their staff and to ensure a workplace free from hazards that may harm employees or cause their death.

Faced with this epidemiological catastrophe, the world is still fighting to control the pandemic. It is necessary to understand the influence of the antecedents from the perspective of the quarantine hotel workers' mental health, especially the need to be empirically explored further in future studies, for instance, whether the hotel employees' knowledge of the pandemic is adequate and what kind of effective strategies, such as providing a communication pathway and counseling service, can relieve their fear and perception of the severity of COVID-19. Through understanding the protective and risk factors of working in the quarantine hotel, policy makers and hoteliers will be able to build a holistic practical and theoretical model to support the hotel employees' mental health and behaviors during the global emergent public health crisis.

## Implications

The basic of hotel service quality depends on the performance of the staff that provides enthusiasm and courtesy service to customers. The staff is a significant asset and treasure of the hotel industry. In fact, the hospitality industry is accepted to be highly stressful environments because of long working hours and night shifts and weekend shifts parameter. During the COVID-19 pandemic, the mental health concern of the quarantine staff who has faced unprecedented changes is undoubtedly necessary. As a significant role of the lodging industry, protecting the staff's mental health has become an urgent task for the hoteliers. To take care of the mental health of these quarantine staff is a critical part of the public health response and also a positive CSR way. While this study drives the quarantine hoteliers' concern about the staffs' mental health in the short term, researchers need to explore and discover effective care strategies of staff mental health, which will bring beneficial CSR to their employees for long.

## Author Contributions

All authors listed have made a substantial, direct and intellectual contribution to the work, and approved it for publication.

## Conflict of Interest

The authors declare that the research was conducted in the absence of any commercial or financial relationships that could be construed as a potential conflict of interest.
